# Pomelo Peel Modified with Citrate as a Sustainable Adsorbent for Removal of Methylene Blue from Aqueous Solution

**DOI:** 10.3390/molecules23061342

**Published:** 2018-06-03

**Authors:** Yimei Ren, Chang Cui, Pengjie Wang

**Affiliations:** 1 Beijing Advanced Innovation Center for Food Nutrition and Human Health, College of Food Science and Nutritional Engineering, China Agricultural University, 17 Qinghuadong Road, Beijing 100083, China; renyimei123@cau.edu.cn; 2 Key Laboratory of Bio-Based Material Science and Technology of Ministry of Education, Northeast Forestry University, 26 Hexing Road, Harbin 150040, China; 15114532032@163.com

**Keywords:** pomelo peel, modification, citric acid, adsorption, methylene blue

## Abstract

An anionic adsorbent was prepared by grafting citrate onto pomelo peel (PPL) to remove methylene blue (MB) from aqueous solution. The PPL and modified pomelo peel (MPPL) were analyzed by Fourier transform infrared spectroscopy (FTIR) and observed by scanning electron microscopy (SEM). The effects of dye concentration, contact time, and pH on adsorption were studied. The FTIR results confirmed that the carboxyl groups were successfully bound to cellulose molecules in PPL via modification with citrate. SEM indicated that the surface of PPL became clean and the porous structure was well maintained after modification. The adsorption capacities of MB onto PPL and MPPL were 81.7 mg/g and 199.2 mg/g, respectively, thus indicating that the addition of anionic groups significantly improved the adsorption performance. The increase in the initial dye concentration and pH of the dye solution promoted the adsorption process. The adsorption equilibrium on MPPL required approximately 3 h. The adsorption of MB on MPPL was well described by a pseudo-second order kinetic model and Langmuir isotherm model. The thermodynamic parameters indicated spontaneous and exothermic adsorption. This study suggests that PPL modified with citrate can be used as a sustainable adsorbent in wastewater purification.

## 1. Introduction

In the past several decades, dye has been commonly used in the textile, paint, paper, food, leather, and rubber industries [1]. Owing to exponential population growth and consumer demand for fashion, dye demand is significantly increasing. Therefore, an increasing amount of dye is released into water already containing high concentrations of complex dyes, thus potentially severely polluting the environment and affecting human health. In the textile industry, approximately 20–30% of the applied dyes are released into effluent because of limited dyeing rates [2]. Therefore, dyes must be removed from wastewater before it enters water bodies.

Compared with physicochemical technologies, adsorption is a feasible and preferred method to completely remove dyes from water, because it is effective toward different types of dyes. In the adsorption process, the adsorbent plays a key role in the separation of pollutants from wastewater. In recent years, low-cost biomass adsorbents [3,4,5] have attracted research attention because of their renewability [6]. Examples of such materials include breadnut peel [7], rice hulls [8], willow branches [9], peanut hulls [10], rice straw [11], bagasse [12], hemp cellulose [13], orange peel [14], and other biomass wastes. The hydroxyl, phenolic, and carboxyl functional groups in the molecular structure of biomass wastes can become active sites for the adsorption of dye wastewater [15,16,17]. In many studies, the adsorption capacity of the biomass itself has been investigated in dye wastewater. Moreover, chemical modification can markedly improve the adsorption capacity of some biomass adsorbents.

Pomelo is planted in China and Southeast Asia; it belongs to the Rutaceae family [18] and is the largest citrus fruit, having a diameter of up to 30 cm. The thick and soft peel accounts for 15% of the entire fruit. High consumption of pomelo produces large amounts of pomelo peels (PPLs), which are disposed of as waste, thus resulting in inefficient resource and land use. PPLs are a lignocellulose biomass mainly composed of cellulose, hemicellulose, and lignin. In some previous studies, PPL has been directly used as an adsorbent for purification of oil pollution [19] and dyes [20]. Other studies have focused on the properties of activated carbon from PPLs [21], including adsorption of uranyl [22], hexavalent chromium [23], and basic dyes [24]. Methylene blue (MB) is extensively used in dyeing materials such as fiber, paper, and hair. Therefore, it is a suitable candidate to demonstrate the application potential of a biomass absorbent. PPL was found to be an attractive candidate for MB adsorption from aqueous solution.

In this study, an eco-friendly and low-cost adsorbent was prepared by grafting citrate onto PPL and was used to remove MB from aqueous solution. The PPL before and after modification (MPPL) was analyzed by Fourier transform infrared spectroscopy (FTIR) and observed by scanning electron microscopy (SEM). The effects of the initial concentration, pH of the MB solution, and contact time on the adsorption of MB were studied. Adsorption kinetics models and isotherms models were used to describe the adsorption process. The thermodynamics of the adsorbents was also investigated.

## 2. Results and Discussion

### 2.1. FTIR Analysis

**Figure 1:**
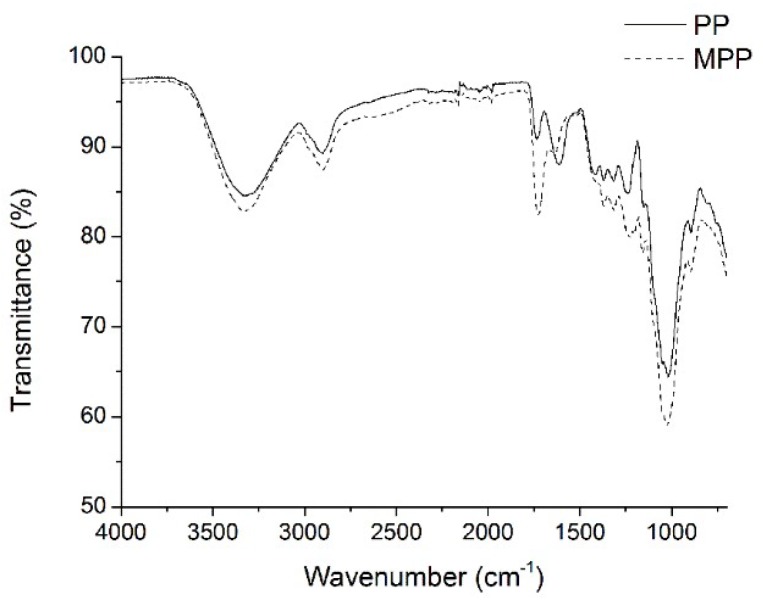
FTIR of the PPL and MPPL.

The FTIR spectra of PPL and MPPL are shown in Figure 1. Characteristic peaks of PPL in the spectrum were observed at 3329 cm^−1^ (–OH stretching vibrations), 2912 cm^−1^ (C–H stretching vibrations), 1733 cm^−1^ (–C=O stretching vibrations in polygalacturonic acid), 1613 cm^−1^ (–C=O stretching vibrations in lignin), and 1239 cm^−1^ (CO-OR stretching vibrations in hemicellulose), and a series of bands were observed at 1152, 1102, 1043, and 1022 cm^−1^, corresponding to –C–O–C-bonds in the anhydroglucose unit of the cellulose or hemicellulose molecule. The results showed that PPL contained cellulose, hemicellulose, lignin, and pectin. After modification, new peaks occurred at 1758 cm^−1^ and 1521 cm^−1^ and were attributed to the ester groups and carboxylate groups, respectively [25]. The changes indicated that carboxylic anions were successfully introduced into the PPL molecule after modification with citrate [26].

### 2.2. SEM Observations

**Figure 2:**
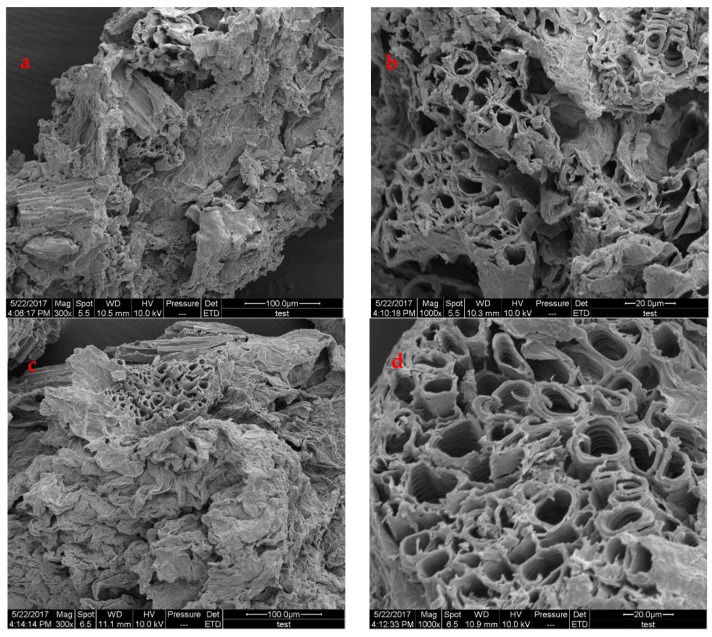
Surface morphology of the PPL (**a**,**b**) and MPPL (**c**,**d**).

The morphology of the PPL before and after modification is shown in Figure 2. The surface of the PPL particle was rough, as shown in micrographs at low magnification. Under magnification, a porous and honeycomb-like structure was clearly seen, which is beneficial for the adsorption of dye. After modification, the porous structure was maintained, thus indicating that the initial structure of PPL was not damaged in the modification process. Moreover, the surface became cleaner after modification because some small impurities were removed in the treatment in citric acid solution.

### 2.3. Comparison of MB Adsorption on PPL and MPPL

The comparison of MB adsorption on PPL before and after modification is shown in Table 1. Under the same experimental conditions, the adsorption capacities of MB on PPL and MPPL were 81.71 mg/g and 199.29 mg/g, respectively. The results indicated that the modification effectively improved the adsorption performance of PPL, owing to the introduction of carboxylic anions, which interacted with cationic dye molecules via electrostatic attraction.

### 2.4. Effect of Initial Concentration on MB Adsorption

**Figure 3:**
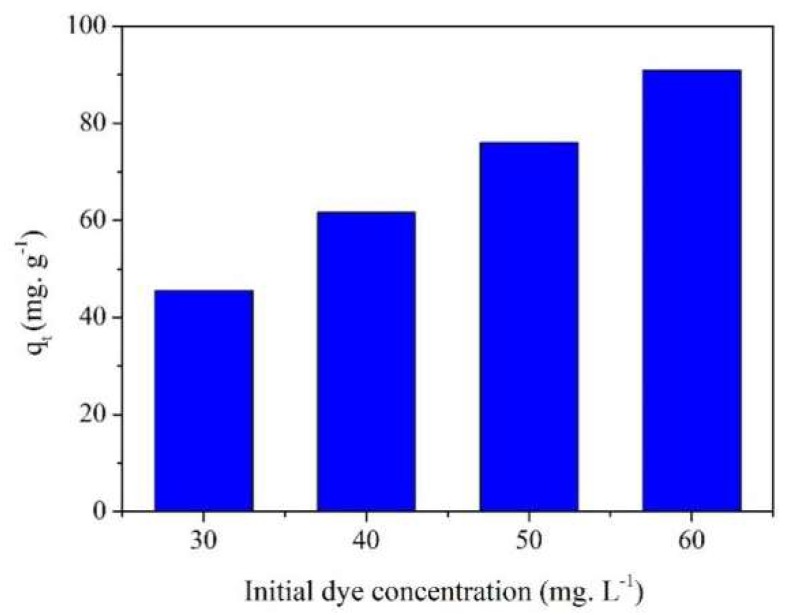
Effect of initial dye concentration on MB adsorption onto MPPL.

Dye concentration is a key factor affecting adsorption. Higher dye concentrations may promote absorption of more dye molecules to the active sites. The effect of initial MB concentration on the adsorption of MB on MPPL is shown in Figure 3. The adsorption capacity increased with increasing MB concentration. The positive correlation between MB concentration and adsorption capacity indicated that the absorbent did not reach saturation under the MB concentrations in the experiments.

### 2.5. Effect of Contact Time on MB Adsorption

**Figure 4:**
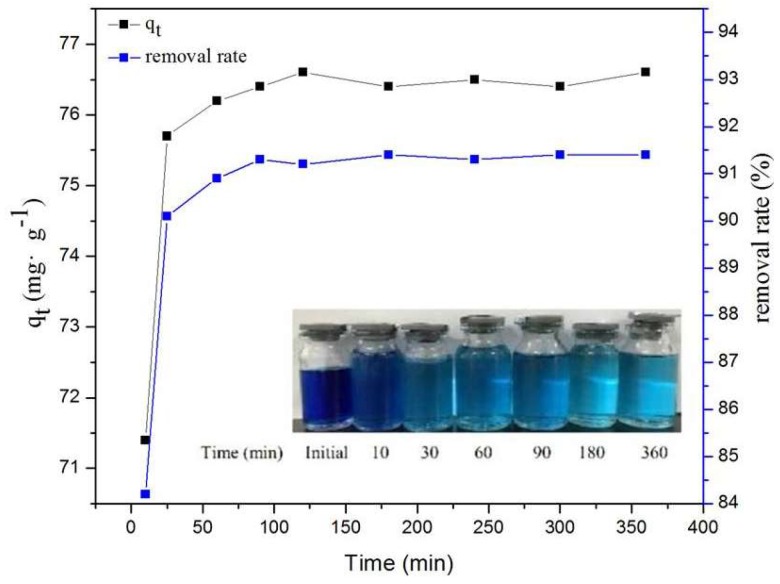
Effect of contact time on MB adsorption onto MPPL.

The effect of contact time on the adsorption of MB on MPPL is shown in Figure 4. The adsorption capacity significantly increased in the first 0.5 h, and the increment of increase decreased as the contact time increased from 0.5 h to 2 h until adsorption equilibrium was reached after 3 h. The initial high adsorption rate may have been due to the higher driving force accelerating molecules to the surface of MPPL and interacting with numerous active sites. The decrease in the adsorption rate may be attributed to the decreased number of active adsorption sites and the long-range diffusion effect of MB molecules. The equilibrium adsorption capacity was up to 45.4 mg/g, and the removal rate was 90.7%. Compared with that of the initial MB solution, the color clearly became lighter after contacting MPPL for 3 h.

### 2.6. Effect of Initial pH on MB Adsorption

**Figure 5:**
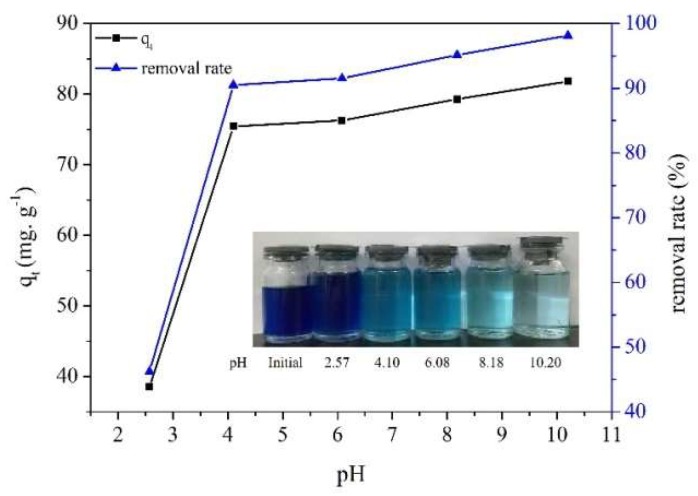
Effect of pH on MB adsorption onto MPPL.

**Figure 6:**
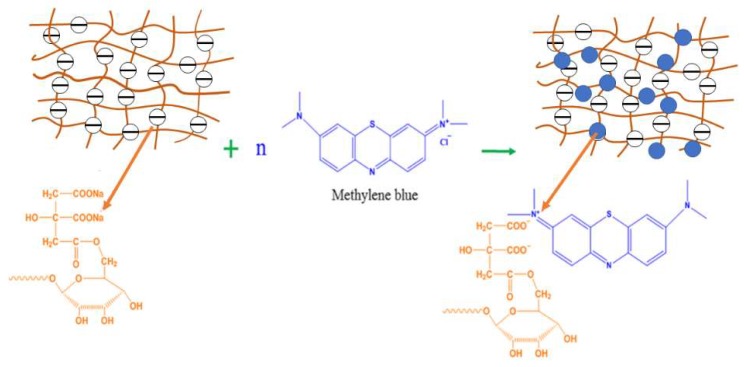
Possible adsorption mechanism for the adsorption of MB on MPPL.

The effects of pH values on the adsorption capacity of MB on MPPL are shown in Figure 5. When the pH value of the MB solution increased from 2.57 to 4.1, the adsorption capacity of MB onto MPPL and the removal rate of MB increased from 38.52 mg/g to 75.43 mg/g and from 46.22% to 90.51%, respectively. A possible adsorption mechanism for the adsorption of MB on MPPL is shown in Figure 6. The pH_PZC_ of MPPL was around 5.3, indicating the surface of MPPL was positively charged when pH <5.3, and the MPPL presented a negative surface when pH >5.3.At lower pH values (pH < 5.3), some of the –COO^−^ groups transformed into –COOH groups, which decrease the electrostatic attraction between the –N^+^ (CH_3_)_2_ Cl^−^ groups in MB molecules and the –COO^−^ groups on MPPL [27]. With increasing pH (pH > 5.3), the adsorption mechanism was mainly controlled by the electrostatic attraction, thus increasing the adsorption capacity because of the higher negative charge on the MPPL surface. In theory, competition of excess OH^−^ ions with the –COO^−^ groups would decrease the adsorption. However, the competition from the OH^−^ ions could decrease the electrostatic attraction between MB molecules and MPPL. Therefore, the adsorption capacity changed slightly when the pH was in the range of 8–10. The inset photograph shows that the solution became almost colorless at pH 10, thus indicating that high pH favored the adsorption of MB on MPPL. The results showed that MPPL is an effective adsorbent for removal of MB from solutions.

### 2.7. Adsorption Kinetics

In the adsorption process, the behavior was determined by the spread of the adsorbate to the adsorbent and by the adsorption rate of the adsorbate on the surface of the adsorbent. Two kinetic models are commonly used to describe the adsorption behavior of dye molecules from aqueous solution on to the adsorbent: pseudo-first order and pseudo-second order kinetic models.

The pseudo-first order kinetic model [28] can be expressed as follows:

ln(q_e_ − q_t_) = lnq_e_ − k_1_t (1)

where q_e_ and q_t_ are the adsorption capacity (mg/g) at equilibrium and time t, respectively, and k_1_ is the rate constant (L min^−1^) of the pseudo-first order kinetic model.

The pseudo-second order kinetic model [29] can be expressed as:

t/q_t_ = 1/k_2_q_e_^2^ + t/q_e_ (2)

where k_2_ (g mg^−1^ min^−1^) is the rate constant for the pseudo-second order kinetic model. The q_e_ and k_2_ values were obtained from the slope (1/q_e_) and intercept (1/k_2_q_e_^2^) of a linear plot of t/q_t_ versus t at different contact times.

**Figure 7:**
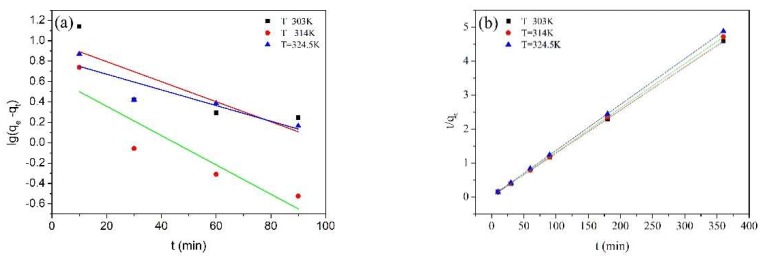
Pseudo-first-order (**a**) and pseudo-second order (**b**) kinetic models of adsorption of MB onto MPPL.

The plots and parameters of kinetic model for MPPL are shown in Figure 7 and Table 2. The correlation coefficients (R^2^) of the pseudo-second order model were more uniform and higher than those of the pseudo-first order model. Moreover, the calculated adsorption capacity (q^e^ calculated) from the pseudo-second order model was closer to the experimental adsorption capacity (q^e^ experimental). The results suggested that the adsorption of MB on MPPL fitted the pseudo-second order model better than the pseudo-first-order model. This indicated that the rate of adsorption process was controlled by chemical process.

### 2.8. Adsorption Isotherms

The Langmuir and Freundlich isotherms are the most often-used isotherm models. The Langmuir isotherm is based on the hypothesis that uptake occurs on a homogeneous surface through monolayer adsorption without interaction between the absorbed molecules [30].

C_e_/q_e_ = 1/(bq_m_) + C_e_/q_m_ (3)

where q_e_ (mg g^−1^) is the equilibrium adsorption capacity of the sorbent; C_e_ (mg L^−1^) is the equilibrium concentration of the dye solution absorbed; qm (mg g^−1^) is the maximum monolayer capacity of the sorbent; and b (L mg^−1^) is the Langmuir isotherm constant.

The essential characteristic of the Langmuir isotherm can be represented by the equilibrium parameter, R_L_, calculated by:

R_L_ = 1/(1 + bC_0_) (4)

where b is the Langmuir constant, and C_0_ is the initial dye concentration (mg/L). R_L_ is a dimensionless separation factor used to determine whether the adsorption process is favorable or unfavorable. The shapes of the isotherms for 0 < R_L_ < 1, R_L_ > 1, R_L_ = 1, and R_L_ = 0 are favorable, unfavorable, linear, and irreversible [1], respectively.

The Freundlich isotherm [31] is given by the following equation:

q_e_ = k_f_C_e_^1/n^ (5)

where k_f_ is the coefficient for the adsorbed amount, and n is the Freundlich constant.

The adsorption data were also found to fit the linear form of the Freundlich equation (R^2^ = 0.990):

lnq_e_ = ln k_f_ + (1/n)lnC_e_ (6)

The calculated parameters from the Langmuir and Freundlich isotherms for MB on MPPL at 30 °C are shown in Table 3. The R^2^ Langmuir value was 0.998 higher than the Freundlich value, thus indicating that the adsorption of MB on MPPL fitted the Langmuir model well. Furthermore, the RL of 0.23 was in the range of 0–1, thus suggesting that the adsorption of MB on MPPL is favorable.

### 2.9. Thermodynamic Study

The adsorption capacity of MB at equilibrium at 303 K, 313 K, and 323 K was used to obtain thermodynamic parameters [32]. Changes in standard enthalpy (ΔH°), standard entropy (ΔS°), and standard Gibbs free energy (ΔG°) were calculated according to the following equations:

ΔG° = –RT ln K_d_ (7)

K_d_ = q_e_/C_e_ (8)

ln K_d_ = –ΔH°/RT + ΔS°/R (9)

where R is the universal gas constant (8.314 J K^−1^ mol^−1^), and K_d_ is the equilibrium constant for the adsorption at standard temperature and pressure.

As shown in Table 4, the ΔG° values were negative, thus indicating that the adsorption of methylene blue dye on MPPL was a spontaneous and favorable process under the experimental conditions. ΔS° was positive, thus indicating increased randomness at the interface of MPPL/MB solution during the adsorption process. The negative ΔH° indicated that the adsorption process was exothermic, and increasing the temperature would not favor the adsorption of MB on MPPL. In addition, an increase in ΔG° value with increasing temperature confirmed that the adsorption of MB on MPPL was more favorable at lower temperature.

## 3. Materials and Methods

### 3.1. Materials

PPL was collected from a local market as solid waste. Citric acid (CA, C_6_H_8_O_7_•H_2_O) purchased from Kemiou Chemical Reagent Co., Ltd. (Tianjin, China) was used as a modification agent for the preparation of adsorbent. MB (C_16_H_18_ClN_3_S) was supplied by Aladdin Chemistry Co. Ltd. (Shanghai, China). All other chemicals and reagents were of analytical grade and were used without further purification.

### 3.2. Preparation of Adsorbents

The yellow skin was removed from pomelo peel, and the white soft and spongy part was obtained and washed several times with distilled water, then air-dried to a constant weight. The pretreated PPL was boiled in distilled water for 1 h to remove some soluble components and then dried at 60 °C. Finally, the dry PPL was ground into small particles (60–80 mesh size).

**Figure 8:**
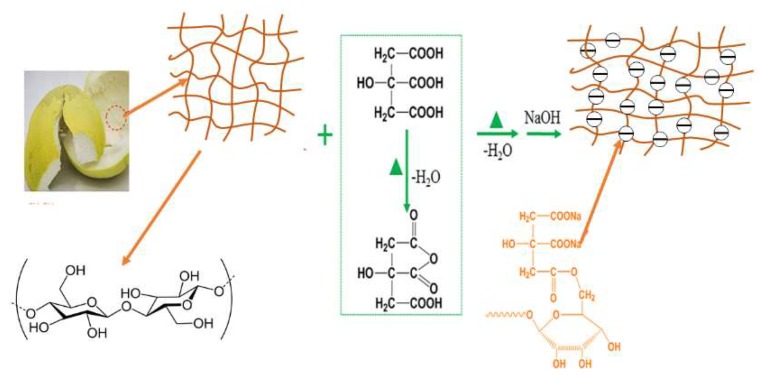
Schematic diagram for the MPPL preparation.

The procedure of the dehydration reaction of citric acid and its reaction with cellulose has been reported [33]. Citric acid first forms a five-membered cyclic anhydride intermediate at elevated temperatures by the dehydration of two adjacent carboxyl groups. The anhydride intermediate then reacts with cellulosic hydroxyl to form an ester linkage. To prepare MPPL, PPL powder (5 g) and 150 mL of 0.6 mol L^−1^ citric acid solution were added to a beaker and stirred at room temperature; the mixture was dried at 55 °C for 20 h and then heated to 120 °C for 90 min. After cooling, the residue was filtered and then immersed into the 0.1 mol L^−1^ of NaOH solution (100 mL) for 1 h. Finally, the sample was washed with hot water at 70 °C and dried in an oven at 60 °C for 24 h. A schematic diagram for MPPL preparation is shown in Figure 8.

### 3.3. Characterization

FTIR spectra were recorded by a TENSOR27 instrument, using the attenuated total reflectance method [34]. The samples were loaded into a KBr disk, followed by extruding the material with 8MT pressure bench press. The FTIR spectrum was measured in the wavenumber of 4000 to 500cm^−1^ at 4 cm^−1^ resolution. Scanning electron microscopy (SEM) (Model Hitachi S-3000N) was applied to observe the morphology of the adsorbent at an electron acceleration voltage of 20 kV. Before measurement, the samples were coated with a sputter coater to make them conductive [35]. The point of zero charge (pH_PZC_) of the adsorbent was determined between pH 2.5 to 11. Then, the initial and final pH of the adsorbent dispersions after storage for 240 min were recorded.

### 3.4. Batch Experiments

Batch experiments were performed to evaluate the performance of the adsorbent in removing MB from aqueous solution [34,35]. In each adsorption experiment, a certain amount of adsorbent and 50 mL of MB solution were mixed into a 250 mL of beaker flask and shaken at 100 rpm. The effects of the initial MB concentration, contact time and pH on the adsorption performance were examined. The pH of the initial MB solution was adjusted with 0.1 mol L^−1^ of NaOH solution and 0.1 mol L^−1^ of HCl solution in the range of 2 to 10, as monitored with a pH meter. After adsorption, the mixture was filtered to remove the adsorbents, and the residual MB solution was measured with a visible spectrophotometer at λ_max_ = 665 nm. The standard curve of MB is expressed by the following equation:

Y = 0.1705X + 0.01506, R^2^ = 0.9984 (10)

The removal rate (R, %) and the adsorption capacity (q, mg g^−1^) were calculated using the following equations:

R = (C_0_ − C_t_)/C_0_ × 100% (11)

q = (C_0_ − C_e_) × V_0_/m (12)

where C_0_, C_e_ and C_t_ (mg L^−1^) are the MB concentration of the residual solution in the initial solution, at equilibrium and at time t, respectively. V_0_ is the volume of MB solution, and m is the mass of the adsorbent.

## 4. Conclusions

In this study, a PPL-based bio-sorbent was prepared through a simple heat esterification method. FTIR analysis revealed that carboxylic anions were successfully introduced to PPL after modification with citrate. The SEM micrographs showed that the modification did not destroy the initial porous structure of PPL. The modification significantly improved the adsorption of MB. The adsorption of MB on MPPL initially increased quickly and reached equilibrium at 3 h. Kinetic analysis indicated that the adsorption was well described by a pseudo-second order model. The adsorption process was well fitted by the Langmuir model, and the predicted maximum adsorption capacity was 104.17 mg/g. The negative values of ΔG°, ΔH°, and ΔS° calculated from the thermodynamic equations indicated that the adsorption of MB on MPPL was spontaneous and exothermic and was an entropy decreasing process. Because of the abundance and wide availability of PPL, and its ease of modification, MPPL may be a potential adsorbent for purification of wastewater containing cationic pollutants.

## Figures and Tables

**Table 1:** Comparison of MB adsorption on PPL before and after modification.

Adsorbent	Dose (mg)	MB Concentration (g/L)	Solution Volume (mL)	Adsorption Capacity (mg/g)
PPL	10	50	50	81.71
MPPL	10	50	50	199.29

**Table 2:** Kinetic parameters for MB dye adsorption onto MPPL.

Temperature (K)	303	314	324.5
q_e_ experimental (mg/g)	78.45	76.50	73.76
pseudo-first-order model			
k_1_ (1/min)	0.029	0.016	0.009
q_e_ calculated (mg/g)	1.64	2.31	2.36
R^2^	0.9747	0.9913	0.9255
pseudo-second-order model			
k_2_ (mg/(g•min))	0.014	0.019	0.018
q_e_ calculated (mg/g)	78.74	76.34	74.07
R^2^	1.0000	1.0000	0.9999

**Table 3:** Isotherm parameters for MB dye adsorption onto MPPL.

Isotherms	Langmuir Model				Freundlich Model		
	b	q_m_	R^2^	R_L_	k_f_	1/n	R^2^
Parameters	0.066	104.17	0.998	0.233	3.289	1.708	0.990

**Table 4:** Thermodynamic parameters for MB adsorption onto PPL and MPPL.

ΔH° (kJ mol^−1^)	ΔS° (J mol^−1^K^−1^)	ΔG° (kJ mol^−1^)		
		303 K	314 K	324.5 K
−27.91	−64.70	−8.30	−7.59	−6.91
